# Chemical Considerations on the Effect of Mordicants, in Dying Cotton Red

**Published:** 1799-04

**Authors:** J. A. Chaptal


### *Chemical Considerations on the Effect of Mordicants, in Dying Cotton Red:—By* J. A. CHAPTAL.

Extracted from his late Memoir read on this Subject, in the National Institute, at Paris.

THE fine red given to cotton, by means of madder, seems to be involved in an uncertainty similar to that prevailing in certain pharmaceutical preparations, the odd and complicated recipes of which have been hitherto respected from an ill-founded notion entertained by the mechanical operators, that the smallest change introduced into the process would destroy the general effect.

A whole month's labour is scarcely sufficient to terminate the different operations thought necessary to obtain the fine Turkey red, called *Adrianople*. For this purpose, a number of different substances are successively employed, soda, oil, gall, sumach, sulphat of alum, blood, madder, soap, the nitro-muriate of tin, &c.

The true method of simplifying this process is not by working at hazard, and attempting, without sufficient guides and experience, a practice different from that in common use. Chemistry is now so rapidly advancing in improvements, that all its operations may be reduced to simple principles, which, when once established, will become, in the hands of the artist, what axioms are to the mathematician.

The three principal *mordicants* in dying cotton red, are, *oil, galls,* and *alum.* To take the red of madder, cotton should be duly impregnated with oil.

oil. This preparation is made by combining oil with a weak solution of soda. The effect of this alkaline lye is merely to dilute, divide, and spread the oil uniformly over all the parts of the cotton. Pot-ash, however, will produce the same effect as the soda, which is an object of some consideration in northern countries, where the latter is scarce and dear, while pot-ash is very common. Yet the soda should be in a caustic state, and contain some of the muriate; this causticity is the effect of its proper calcination.

In the choice of the oil great care should be taken, as well as in that of the soda. The oil used for painting is the most proper; not the finest oil, but that which contains a strong portion of the *extractive principle*. It ought, moreover, to be in excess, that is, not in a state of absolute saturation with the alkali.

When the cotton has been sufficiently impregnated with oil, it is then subjected to the operation of *galling*; here galls are of great service, and cannot be replaced by any other astringent vegetable substances, whatever quantities of them may be employed.

The third mordicant necessary, in the dying of cotton red, is the sulphat of alum. It not only possesses the property of giving a gloss to the red of madder, but it contributes also, by its decomposition, and the fixation of its aluminous base, to give solidity to the colour. To judge of the effects of alum in dying cotton, it may suffice to mix a decoction of galls, with a solution of alum. This mixture instantly becomes turbid, and a greyish precipitate is formed, which, when dried, is insoluble in water and in alkalies.—The solution of alum, however, should not be too hot, as in that case a portion of the astringency obtained from the galls will escape, from the texture of the cotton, and then the decomposition of the alum will take place in the immersion; a circumstance which must necessarily diminish the effect of the mordicant, and contribute to impoverish the colour.

Here then is a combination of three principles, viz. oil, the astringent principle, and the base of alum, which produce the united effects as a mordicant to the red of madder. If separately employed, they will neither be attended with the same fixidity, nor afford a similar gloss and brightness in the colour.

This mordicant is unquestionably the most artificial that is known in dying, and the operation of fixing the red colour in cotton is also the most complicated of all others. It presents to the chemist a species of combination, highly interesting in the prosecution of his studies.

Experience alone is ſufficient to conduct us in theſe numerous operations and experiments: we ought to reaſon upon them, and calculate both the principle and reſult of each, without which, the labours of the moſt experienced operator, far from enabling him to correct errors, and obtain uniform products, will only preſent a diſcouraging alternation of ſucceſſes and failures.

